# Inverse Ni/CeCrO_x_ Catalysts for Enhanced Low-Temperature CO_2_ Methanation

**DOI:** 10.3390/ijms27073193

**Published:** 2026-03-31

**Authors:** Da Zhang, Haiyu Qi, Bowen Lei, Xuan Guo, Feiyan Fu

**Affiliations:** State Key Laboratory of Chemistry for NBC Hazards Protection, Beijing 102205, China; fireulc@163.com (D.Z.); haiyuqi2001@126.com (H.Q.); lbw3180@163.com (B.L.)

**Keywords:** CO_2_ hydrogenation, inverse catalyst, nickel, low-temperature methanation

## Abstract

Low-temperature methanation technology offers a promising pathway for carbon recycling and sustainable energy storage by enabling near-equilibrium CO_2_ conversion under atmospheric pressure. However, efficiently activating CO_2_ at low temperatures remains a significant challenge due to the kinetic limitations of hydrogenation intermediates. We construct a composite oxide–metal interface structure by anchoring highly dispersed CeCrO_x_ nanoclusters onto metallic nickel via an ion-exchange method. This catalyst exhibits superior activity compared to conventional Ni/oxide catalysts with identical composition. Under atmospheric pressure at 220 °C, it achieves nearly 80% CO_2_ conversion with over 99% methane selectivity and maintains excellent catalytic performance and structural stability during a 240-h continuous test. Systematic characterizations, including high-resolution transmission electron microscopy, X-ray photoelectron spectroscopy, CO_2_ temperature-programmed desorption, and in situ DRIFTS reflectance infrared Fourier-transform spectroscopy, reveal that the synergistic modification by CeO_2_ and Cr_2_O_3_ not only optimizes the electronic structure of Ni to promote CO_2_ adsorption and activation, but also enhances H_2_ dissociation and intermediate conversion by regulating oxygen vacancy concentration and alkaline site distribution. Mechanistic studies indicate that the reaction follows a synergistic mechanism dominated by the formate pathway and assisted by the CO pathway. Moreover, the interfacial structure effectively stabilizes active sites and inhibits carbon deposition from CH_4_ decomposition. This study provides a universal and effective strategy for designing Ni-based CO_2_ conversion catalysts suited for mild reaction conditions and characterized by high energy efficiency.

## 1. Introduction

The rising atmospheric concentration of carbon dioxide (CO_2_), a primary greenhouse gas, driven by global economic growth and reliance on fossil fuels, has spurred intensive research into carbon capture and utilization (CCU) technologies [1,2,3]. Among various CCU conversion pathways, CO_2_ hydrogenation to methane (CO_2_ + 4H_2_ → CH_4_ + 2H_2_O) stands out due to its higher industrial feasibility compared to alternatives like methanol synthesis [4,5,6]. This process offers advantages including mild reaction conditions, high CO_2_ conversion efficiency, and direct compatibility with existing natural gas infrastructure, serving a dual role in CO_2_ emission reduction and hydrogen storage [7]. Despite its thermodynamic favorability, the kinetics of CO_2_ methanation remain a limiting factor [8,9]. Conventional nickel-based catalysts, while cost-effective and selective, typically require temperatures exceeding 300 °C to achieve practical reaction rates. This often leads to catalyst sintering and deactivation via carbon deposition [10,11,12]. Developing highly active, sintering-resistant low-temperature catalysts thus represents a central challenge in heterogeneous catalysis.

Historically, noble metal catalysts (e.g., Ru, Rh) have demonstrated high intrinsic activity and stability for low-temperature methanation (around 250 °C), but their scarcity and high cost limit large-scale application [13,14]. Research focus has consequently shifted to non-precious alternatives, with nickel-based systems receiving particular attention due to their activity and abundance. A common strategy to enhance low-temperature (230–250 °C) activity involves increasing nickel loading (40–50 wt%); however, optimal performance still requires temperatures above 200 °C [15,16,17]. This limitation is often attributed to insufficient reactivity at conventional nickel–oxide interfaces, which fail to effectively stabilize and hydrogenate key oxygenated intermediates (e.g., COOH, HCOO).

To address this, engineering catalyst interfaces has emerged as a promising research direction. Modifying supports such as CeO_2_ (for oxygen storage capacity), ZrO_2_ (for thermal stability), TiO_2_ (for strong metal–support interaction), or Al_2_O_3_ (for high surface area) can improve metal dispersion, redox properties, and overall durability [18,19,20,21]. Recently, the inverse O/M catalyst architecture—dispersing oxide nanoparticles on a metallic nickel matrix—has gained increasing interest. This configuration, originally proposed by G. M. Schwab, who emphasized the electronic effect at the oxide–metal contact interface, can create asymmetric adsorption sites at the oxide–metal boundary [22]. These sites help stabilize active intermediates and promote their hydrogenation. Studies over the past three years indicate that such inverse structures can effectively mitigate the low reducibility and high-temperature requirements of a conventional nickel catalyst [23,24,25]. Nevertheless, key questions regarding the electronic structure of these interfaces, the optimal nickel loading in inverse systems, and their long-term stability under industrial conditions remain insufficiently answered.

This study reports an inverse supported nickel catalyst synthesized via an ion-exchange method, which achieves remarkably enhanced activity for low-temperature CO_2_ methanation. Compared to a conventional Ni/oxide catalyst of identical composition, the inverse catalyst demonstrates superior performance within the 110–200 °C range. The inverse 85 wt% Ni/CeCrO_x_ catalyst achieves a CO_2_ conversion of 69% at 180 °C and exceeds 80% at 220 °C, while maintaining > 99% CH_4_ selectivity and excellent stability during a 240-h test at 220 °C. In situ DRIFTS results reveal that CO_2_ readily dissociates to CO via the RWGS + CO pathway at the Cr_2_O_3_-Ni interface. At the CeCrO_x_-Ni interface, the reaction follows a synergistic mechanism dominated by the formate pathway with the CO pathway as a secondary route. This work provides a universal and effective strategy for designing nickel-based CO_2_ conversion catalysts suited for mild reaction conditions and efficient energy utilization.

## 2. Results and Discussion

### 2.1. Preparation of Inverse Catalysts

The Ni(OH)_2_ nanosheets were prepared via an ion-exchange method. Subsequently, the Ni(OH)_2_ layer was modified with cerium and aluminum oxides through a hydrothermal process followed by calcination at 500 °C (see Methods). The resulting catalyst is denoted as 85 wt% Ni/CeCrO_x_. Other reference catalysts, including 85 wt% Ni/Cr_2_O_3_ and 85 wt% Ni/CeO_2_, were synthesized using the same procedure. Prior to performance evaluation, all catalysts were pre-reduced at 500 °C under a H_2_ flow of 50 mL/min for 2 h to convert the NiO substrate into metallic Ni. For comparison with the Ni/CeCrO_x_ composite catalyst, Ni supported on Cr_2_O_3_ and CeO_2_ oxide supports was also prepared by an identical method, with the Ni loading controlled at 85 wt%. Catalytic performance was evaluated under atmospheric pressure using an identical feed composition and experimental setup (Supplementary Figure S1).

**Figure 1:**
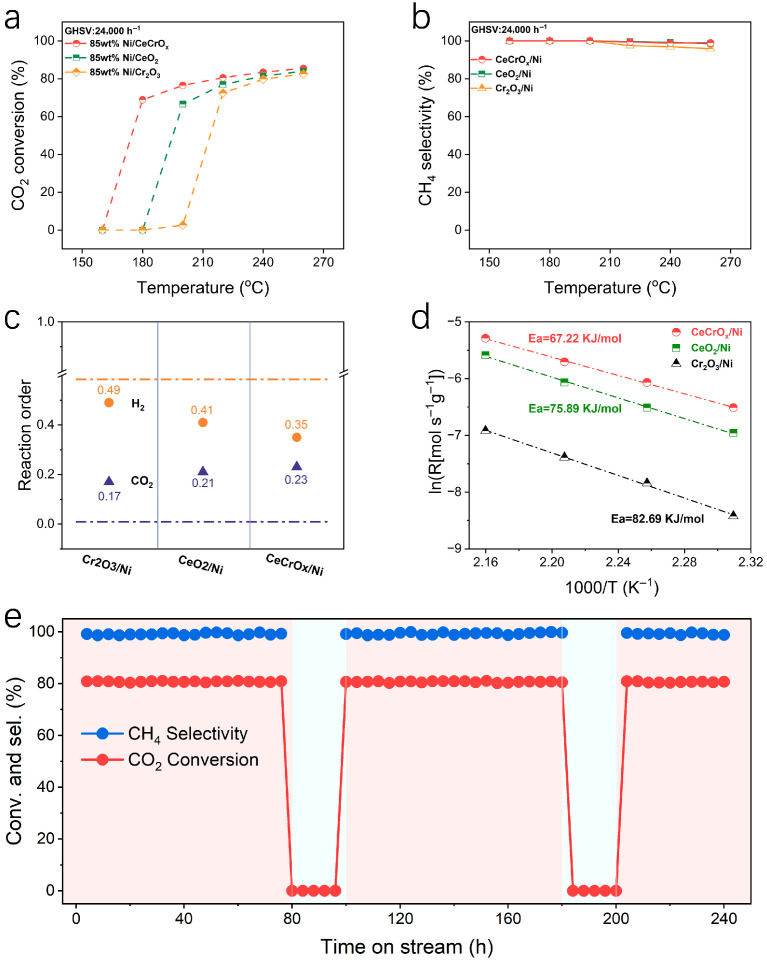
The catalytic performance of inverse catalysts. (**a**,**b**) Temperature-dependent CO_2_ conversion and CH_4_ selectivity of the 85 wt% Ni/CeCrO_x_, 85 wt% Ni/CeO_2_ and 85 wt% Ni/Cr_2_O_3_ catalysts (reaction conditions: GHSV = 24,000 h^−1^, 160–260 °C CO_2_:H_2_:N_2_ = 8:32:10, *p*= 0.1 MPa). (**c**) Reaction orders with respect to H_2_ and CO_2_ for methane formation. (**d**) CH_4_-based apparent activation energy (*E_a_*) of 85 wt% Ni/CeCrO_x_, 85 wt% Ni/CeO_2_ and 85 wt% Ni/Cr_2_O_3_ catalysts. (**e**) Stability test of 85 wt% Ni/CeCrO_x_ catalyst at 220 °C.

Catalytic performance evaluation was conducted in a fixed-bed reactor under atmospheric pressure (0.1 MPa). Typically, 125 mg of the catalyst was pelletized and sieved to 40–60 mesh, and then diluted with quartz sand at a mass ratio of 1:1 (catalyst:sand). The reactant gas mixture consisting of 16% CO_2_, 64% H_2_, and 20% N_2_ was fed at a GHSV of 24,000 mL·g_cat_^−1^·h^−1^. To ensure that the obtained kinetic data were free from heat and mass transfer limitations, dedicated experiments were performed to exclude both external and internal diffusion effects. External diffusion was verified by varying the catalyst particle size while maintaining other reaction conditions constant (16% CO_2_/64% H_2_/20% N_2_, 0.1 MPa, 165 °C, 24,000 mL·g_cat_^−1^·h^−1^); the CO_2_ conversion remained unchanged with different particle sizes, confirming the absence of external diffusion limitation [26] (Supplementary Figure S2). Furthermore, internal diffusion was assessed by testing catalysts with different pellet sizes under identical gas composition and temperature (165 °C), with no variation in activity observed, indicating negligible internal diffusion influence [27] (Supplementary Figure S3). The 85 wt% Ni/Cr_2_O_3_ catalyst achieved 74% CO_2_ conversion at 240 °C, and the 85 wt% Ni/CeO_2_ catalyst reached 77% at 260 °C, whereas the 85 wt% Ni/CeCrO_x_ catalyst exhibited 80% conversion at a lower temperature of 220 °C, highlighting the crucial role of the inverse structure and oxide modification (Figure 1a,b). The formation of a Ce-Cr mixed oxide phase in the 85 wt% Ni/CeCrO_x_ catalyst further reduced the activation temperature to 160 °C. This catalyst showed high performance with about 80% CO_2_ conversion and >99.9% CH_4_ selectivity at 220 °C, substantially surpassing conventional oxide-supported Ni catalysts (Supplementary Figures S4 and S5). The 85 wt% Ni/CeCrO_x_ catalyst demonstrates a CO_2_ conversion of approximately 68.9% at 180 °C, outperforming most reported Ni-based catalysts that typically require significantly higher temperatures to achieve comparable activity, such as 20Ce80Co (58.3% at 200 °C) and CeO_2_/Ni-4 (67.5% at 225 °C) [28,29]. The Ni/La_0.2_-MgAlO_x_ catalyst achieves a CO_2_ conversion of 69% at 225 °C, surpassing the Ni/CeCrO_x_ catalysts’ performance at 180 °C but at a significantly higher temperature [30] (Supplementary Figure S6 and Supplementary Table S1). The catalyst also exhibits excellent stability over 240 h with no obvious deactivation, in contrast to significant activity loss observed for 85 wt% Ni/Cr_2_O_3_ under identical conditions.

To compare the reaction orders of CO_2_ and H_2_, the CO_2_ feed gas was diluted to maintain the reaction within the kinetic regime and to eliminate hotspot effects. Kinetic analysis of the CO_2_ methanation catalysts revealed apparent H_2_ and CO_2_ reaction orders of 0.35 and 0.23 for 85 wt% Ni/CeCrO_x_, 0.41 and 0.21 for 85 wt% Ni/CeO_2_, and 0.49 and 0.17 for 85 wt% Ni/Cr_2_O_3_ (Figure 1c). The shift in these apparent orders indicates that over the MO_x_ ensembles of 85 wt% Ni/CeCrO_x_, CO_2_ surface coverage decreases while H_2_ coverage increases, consistent with the Langmuir–Hinshelwood mechanism [31,32]. This change substantially promotes the surface reaction rate. By adjusting the gas hourly space velocity (GHSV) for each catalyst, all CO_2_ conversions used in the activation energy (*Ea*) calculation were kept below 10%. The apparent activation energy for CH_4_ formation over 85 wt% Ni/CeCrO_x_ was determined to be 67.22 kJ/mol, lower than that of 85 wt% Ni/CeO_2_ (75.89 kJ/mol) and 85 wt% Ni/Cr_2_O_3_ (82.69 kJ/mol) (Figure 1d and Supplementary Figure S7). These results confirm the significant role of the CeCrO_x_/Ni inverse structure in enhancing the reaction kinetics of CO_2_ hydrogenation to methane. The CH_4_ space-time yield (STY) of 85 wt% Ni/CeCrO_x_ and 30 wt% Ni/CeCrO_x_ catalysts was investigated within the kinetic regime (GHSV = 24,000 mL·g_cat_^−1^·h^−1^, CO_2_ conversion < 15% at 150 °C) (Supplementary Figure S8). For the 85 wt% Ni/CeCrO_x_ catalyst, STY increased markedly with temperature from 1.3 mmol g^−1^ h^−1^ at 130 °C to 10.1 mmol g^−1^ h^−1^ at 170 °C. In contrast, the 30 wt% Ni/CeCrO_x_ catalyst exhibited lower STY values across the same temperature range, rising from 0.3 mmol g^−1^ h^−1^ at 130 °C to 6.9 mmol g^−1^ h^−1^ at 170 °C. In the kinetic regime, the 85 wt% Ni/CeCrO_x_ catalyst exhibited a CH_4_ space-time yield (STY) of 17.14 mmol g_cat_^−1^ h^−1^, which is 2.5 times higher than that of the 30 wt% Ni/CeCrO_x_ catalyst. The 85 wt% Ni/CeCrO_x_ catalyst consistently delivered higher STY at all tested temperatures, indicating superior catalytic efficiency per unit mass. This enhanced performance can be attributed to the inverse catalyst architecture with higher nickel loading, which provides a greater density of active sites and promotes CH_4_ formation [22,33].

Usually, supported nickel catalysts tend to deactivate under harsh reaction conditions in CO_2_ methanation (such as overheating and start–stop cycles), mainly due to a strong tendency for sintering and coke deposition [34,35,36]. To investigate the stability of the catalyst under long-term reaction conditions, the 85 wt% Ni/CeCrO_x_ catalyst was tested in a 240-h reaction at atmospheric pressure and 220 °C. During the stability test, two start–stop cycles were performed, with cooling pauses at 80–100 h and 180–200 h. Under a GHSV of 24,000 mL·g_cat_^−1^·h^−1^, the 85 wt% Ni/CeCrO_x_ catalyst maintained approximately 80% CO_2_ conversion and >99% CH_4_ selectivity, with no observable deactivation (Figure 1e and Supplementary Figure S9).

### 2.2. Structural Characterization of the Inverse Catalysts

The Ni(OH)_2_ nanosheets were first prepared via an ion-exchange method, followed by the surface modification with cerium and chromium oxides through a hydrothermal reaction and subsequent calcination at 500 °C (see Methods). The obtained catalyst is labeled as 85 wt% Ni/CeCrO_x_, in which the Ce and Cr loadings, as determined by inductively coupled plasma optical emission spectrometry (ICP-OES), are 2.1 wt% and 2.0 wt%, respectively. The reference catalysts, 85 wt% Ni/CeO_2_ and 85 wt% Ni/Cr_2_O_3_, were synthesized following the same procedure. Prior to performance evaluation, all catalysts underwent pre-reduction in a 50 mL/min H_2_ flow at 500 °C for 2 h to transform the NiO substrate into metallic Ni.

**Figure 2:**
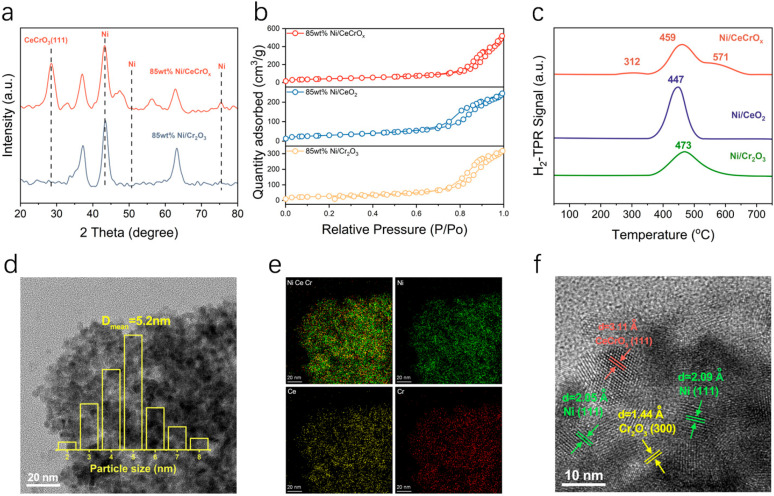
Structural characterization of inverse catalysts. (**a**) XRD patterns of the fresh Ni/CeCrO_x_ and fresh Ni/Cr_2_O_3_ catalysts. (**b**) N_2_ adsorption–desorption isotherms of 85 wt% Ni/CeCrO_x_, 85 wt% Ni/CeO_2_ and 85 wt% Ni/Cr_2_O_3_ catalysts. (**c**) H_2_-TPR profiles of 85 wt% Ni/CeCrO_x_, 85 wt% Ni/CeO_2_ and 85 wt% Ni/Cr_2_O_3_ catalysts before reduction. (**d**) TEM image of 85 wt% Ni/CeCrO_x_ catalyst (inset is the particle size distribution histogram). (**e**) Elemental mappings of Ni, Ce, and Cr elements in 85 wt% Ni/CeCrO_x_ (scale bar, 10 nm). (**f**) High-resolution TEM image of 85 wt% Ni/CeCrO_x_ (scale bar, 2 nm).

The phase structures of the 85 wt% Ni/CeCrO_x_ and 85 wt% Ni/Cr_2_O_3_ catalysts were characterized by X-ray diffraction (XRD). The patterns revealed three intense diffraction peaks at 2θ = 44.50°, 51.85°, and 76.37° for both catalysts, which are attributed to the (111), (200), and (220) crystal planes of metallic Ni, respectively [37,38]. Only these characteristic Ni peaks are observed for the 85 wt% Ni/Cr_2_O_3_ sample (Figure 2a). In contrast, the 85 wt% Ni/CeCrO_x_ catalyst exhibits an additional strong peak at 2θ = 28.7°, which is assigned to the (111) plane of CeCrO_3_. Furthermore, the Ni (111) diffraction peak of the 85 wt% Ni/CeCrO_x_ catalyst is broader than that of the 85 wt% Ni/Cr_2_O_3_ catalyst. This peak broadening indicates that constructing an oxide–metal interface through the introduction of the composite oxide effectively reduces the Ni particle size and enhances its dispersion. All catalysts exhibit type-IV isotherms with H_3_-type hysteresis loops, confirming the presence of a mesoporous structure [39,40]. The specific surface areas of the catalysts were determined using the Barrett–Joyner–Halenda (BJH) method (Figure 2b). The measured values are 149.9 m^2^/g for 85 wt% Ni/CeCrO_x_, 107.6 m^2^/g for 85 wt% Ni/CeO_2_, and 101.9 m^2^/g for 85 wt% Ni/Cr_2_O_3_. The average pore diameters of the catalysts are 21.4 nm, 14.1 nm, and 19.3 nm, respectively (Table 1).

The reducibility and metal–support interactions of the catalysts were investigated by H_2_ temperature-programmed reduction (H_2_-TPR). For the 85 wt% Ni/CeO_2_ catalyst, a single reduction peak centered at 447 °C is observed, which is attributed to the reduction of NiO species that interact moderately with the CeO_2_ support (Figure 2c). For the 85 wt% Ni/Cr_2_O_3_ catalyst, the main reduction peak appears at a higher temperature of 473 °C, indicating a stronger interaction between NiO and Cr_2_O_3_, which makes the NiO species more difficult to reduce. Notably, the 85 wt% Ni/CeCrO_x_ catalyst exhibits three distinct reduction peaks at 312 °C, 459 °C, and 571 °C. The low-temperature peak at 312 °C can be assigned to the reduction of highly dispersed NiO species with weak interaction with the support. The peak at 459 °C is attributed to the reduction of NiO species that interact moderately with the CeCrO_x_ composite oxide. The high-temperature peak at 571 °C suggests the presence of strongly interacting NiO species, possibly associated with the formation of Ni-O-Ce or Ni-O-Cr interfacial structures. Compared to the single-component oxide-supported catalysts, the multi-peak reduction behavior of the 85 wt% Ni/CeCrO_x_ catalyst demonstrates the synergistic effect between CeO_2_ and Cr_2_O_3_, which creates heterogeneous NiO species with different degrees of interaction.

**Figure 3:**
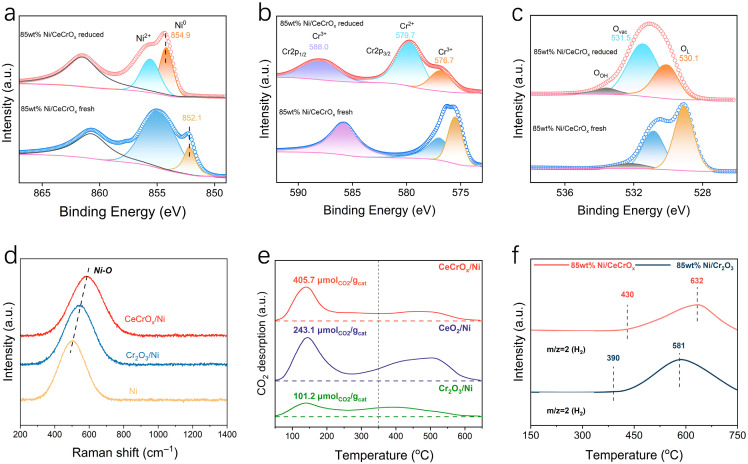
Structural characterization of inverse catalysts. (**a**–**c**) XPS spectra of Ni 2p, O 1s, and Cr 2p regions for reduced 85 wt% Ni/CeCrO_x_ and fresh 85 wt% Ni/CeCrO_x_ catalysts. (**d**) Raman spectra of the Ni, 85 wt% Ni/CeCrO_x_ and 85 wt% Ni/Cr_2_O_3_ catalysts. (**e**) CO_2_-TPD profiles of 85 wt% Ni/CeCrO_x_, 85 wt% Ni/CeO_2_ and 85 wt% Ni/Cr_2_O_3_ catalysts. (**f**) TPSR results of methane on 85 wt% Ni/CeCrO_x_ and 85 wt% Ni/Cr_2_O_3_ catalysts. Reaction conditions: 10 vol% CH_4_/Ar, GHSV = 24,000 h^−1^.

The morphology and microstructure of 85 wt% Ni/CeCrO_x_ were further characterized using electron microscopy. Scanning electron microscopy (SEM) images reveal a uniform distribution of nano-sized oxide particles on the surface of the reduced catalyst. These particles exhibit an island-like morphology and are tightly anchored on the metallic Ni substrate, with a particle size distribution centered around 5.4 nm (Figure 2d). This configuration establishes a well-defined oxide–metal inverse interface, which is anticipated to modulate the local electronic structure and enhance reactant adsorption. High-angle annular dark-field scanning transmission electron microscopy (HAADF-STEM) and energy-dispersive X-ray spectroscopy (EDS) mapping further confirm the uniform dispersion of Ce and Cr elements across the NiO particles in the nanocomposite (Figure 2e). High-resolution imaging shows well-resolved lattice fringes corresponding to a d-spacing of 0.311 nm. This spacing corresponds to the (111) plane of CeCrO_3_, demonstrating the formation of a CeCrO_x_ mixed oxide and its successful loading onto the NiO support (Figure 2f). The chemical state of the catalyst surface was examined by in situ X-ray photoelectron spectroscopy (XPS). The Ni 2p XPS spectra confirm that the calcined 85 wt% Ni/CeCrO_x_ catalyst surface predominantly consists of Ni^2+^ species (>66%), which are converted to metallic Ni^0^ after reduction [41] (Figure 3a and Supplementary Table S2). According to the Ce 3d spectra, approximately 36% of surface Ce atoms exist as Ce^3+^ after calcination, and this proportion increases to over 45% following reduction (Supplementary Figure S10). The increase in Ce^3+^ content promotes the formation of oxygen vacancies within the composite structure [42] (Supplementary Table S3). The Cr 2p XPS spectrum of the reduced 85 wt% Ni/CeCrO_x_ catalyst reveals the coexistence of Cr^3+^ and Cr^2+^ species, with the Cr^2+^ 2p_3/2_ peak appearing at approximately 579.7 eV (Supplementary Table S4). The presence of reduced Cr^2+^ species indicates electron transfer from Ni^0^ to Cr^3+^ via Ni-O-Cr bridging bonds at the oxide–metal interface, verifying the strong electronic interaction within the CeCrO_x_ mixed oxide (Figure 3b). The surface oxygen to total oxygen ratio (O_vac_/(O_OH_ + O_vac_ + O_L_)) reaches approximately 61% for the 85 wt% Ni/CeCrO_x_ catalyst based on O 1s XPS analysis (Figure 3c and Supplementary Table S5), indicating a higher density of oxygen vacancies in the CeCrO_x_ mixed oxide.

We investigated the metal–oxygen vibration modes of different catalysts using Raman spectroscopy. In 85 wt% Ni/Cr_2_O_3_, the Ni-O vibration peak shifts to 552 cm^−1^, exhibiting a redshift compared to that in NiO/Ni (503 cm^−1^) [43] (Figure 3d). This shift likely results from the formation of Cr-O-Ni coordination. When Ce is introduced in the 85 wt% Ni/CeCrO_x_ catalyst, a more pronounced redshift of the Ni-O vibration is observed, without the appearance of a Ce-O vibration peak. These features indicate the formation of a CeCrO_x_ mixed oxide, which subsequently modifies the Ni-O vibration. To investigate the origin of the excellent catalytic performance of the 85 wt% Ni/CeCrO_x_ catalyst, we characterized its active sites using CO_2_ temperature-programmed desorption (CO_2_-TPD). The desorption profiles revealed three distinct regions (50–200 °C, 200–400 °C, and >400 °C), corresponding to weak, medium-strength, and strong alkaline sites, respectively [44]. The weak and medium-strength sites showed the highest reactivity for CO_2_ methanation, with the 85 wt% Ni/CeCrO_x_ catalyst exhibiting particularly high CO_2_ adsorption on weak sites (405.7 μmol/g_cat_)—approximately 1.7 and 4.0 times greater than that of 85 wt% Ni/CeO_2_ and 85 wt% Ni/Cr_2_O_3_, respectively (Figure 3e). Although the total adsorption capacity was not the highest, this enhanced weak-site adsorption provides a basis for its superior CO_2_ conversion [45,46] (Supplementary Table S6). The introduction of Cr_2_O_3_ also significantly reduced strong alkaline sites, likely due to modified electronic distribution of Ni [47]. A confirmed linear correlation between the capacity of weakly/medium-adsorbed CO_2_ and the intrinsic CH_4_ production rates at 160–220 °C indicates that oxygen vacancies at the inverse oxide–metal interface are probable active sites for low-temperature CO_2_ activation, explaining the catalyst’s high methanation activity (Supplementary Figure S11).

### 2.3. The Mechanism of CO_2_ Hydrogenation on Inverse Interface

**Figure 4:**
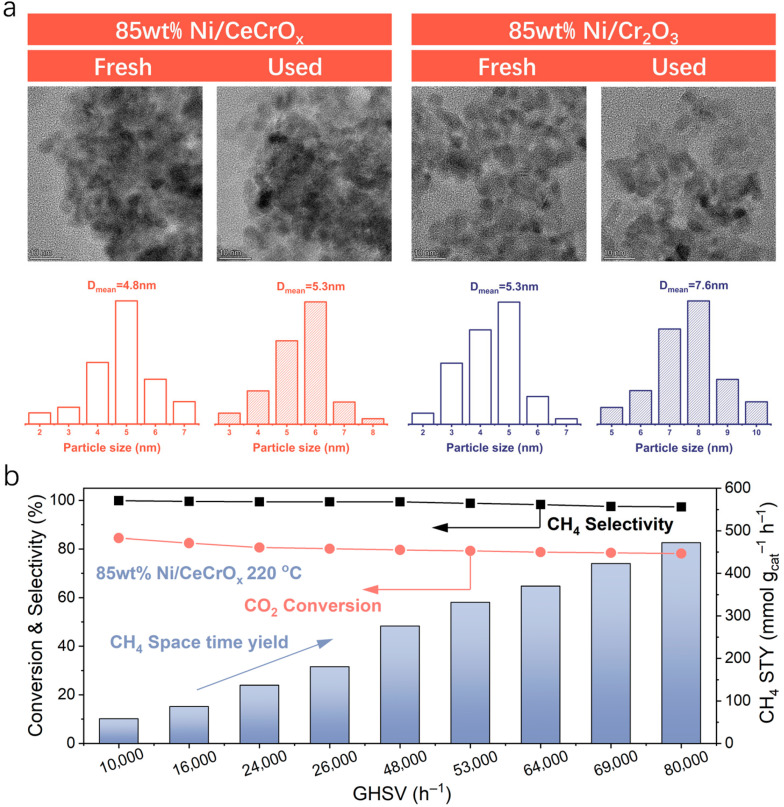
Structural thermal stability and GHSV-dependent activities of 85 wt% Ni/CeCrO_x_ and 85 wt% Ni/Cr_2_O_3_ catalysts. (**a**) STEM images of 85 wt% Ni/CeCrO_x_ and 85 wt% Ni/Cr_2_O_3_ catalysts before and after use. (**b**) GHSV-dependent activities of 85 wt% Ni/CeCrO_x_ catalyst at 220 °C.

Reaction stability is a critical indicator for practical catalysts, particularly for CO_2_ methanation catalysts that face significant challenges of sintering and carbon deposition. A 240-h stability test was conducted on the 85 wt% Ni/Cr_2_O_3_ catalyst at 220 °C. Its catalytic activity declined after experiencing two start–stop cycles. In contrast, the 85 wt% Ni/CeCrO_x_ catalyst showed no significant loss in activity under the same cycling conditions. The decrease in CO_2_ conversion over 85 wt% Ni/Cr_2_O_3_ likely originates from the agglomeration of Ni nanoparticles. This observation indicates that the interaction between the oxide and the Ni substrate can effectively inhibit the migration of Ni species, thereby preventing undesirable sintering. The anti-coking mechanism of the Ni/CeCrO_x_ catalyst was investigated by CH_4_ temperature-programmed surface reaction (CH_4_-TPSR). The onset temperature for CH_4_ decomposition into H_2_ and carbon over this catalyst was approximately 40 °C higher than that over the 85 wt% Ni/Cr_2_O_3_ catalyst (Figure 3f). Furthermore, the CeCrO_x_ inverse species maintained a high degree of dispersion without agglomeration after the stability test, with the particle size only increasing slightly from 4.8 nm to 5.3 nm. Conversely, the Ni nanoparticles on the Ni/Cr_2_O_3_ catalyst sintered from 5.3 nm to 7.6 nm after the start–stop cycles (Figure 4a). These results further confirm that the oxide–Ni interaction effectively suppresses Ni species migration and sintering. At 220 °C, the space-time yield (STY) of CH_4_ was further evaluated as a function of gaseous hourly space velocity (GHSV). The 85 wt% Ni/CeCrO_x_ catalyst maintained CO_2_ conversion above 78% even at a high GHSV of 80,000 h^−1^. Under this condition, the corresponding methane STY reached 472 mmol g_cat_^−1^ h^−1^ with a selectivity exceeding 97% (Figure 4b).

**Figure 5:**
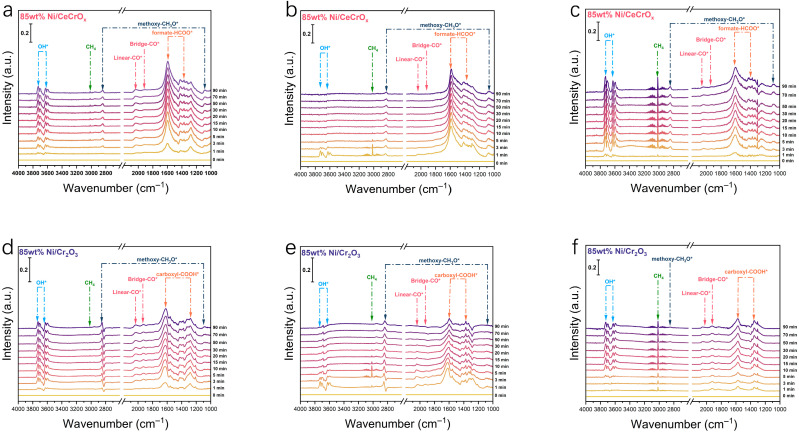
Mechanistic route study. (**a**) CO_2_ adsorption of in situ DRIFTS at 220 °C on 85 wt% Ni/CeCrO_x_ catalyst. (**b**) H_2_ adsorption of in situ DRIFTS at 220 °C on 85 wt% Ni/CeCrO_x_ catalyst. (**c**) CO_2_/H_2_ mixture adsorption of in situ DRIFTS at 220 °C on 85 wt% Ni/CeCrO_x_ catalyst. (**d**) CO_2_ adsorption of in situ DRIFTS at 220 °C on 85 wt% Ni/Cr_2_O_3_ catalyst. (**e**) H_2_ adsorption of in situ DRIFTS at 220 °C on 85 wt% Ni/Cr_2_O_3_ catalyst. (**f**) CO_2_/H_2_ mixture adsorption of in situ DRIFTS at 220 °C on 85 wt% Ni/Cr_2_O_3_ catalyst. * denotes surface adsorbed species.

In situ DRIFTS was employed to investigate the reactive intermediates and mechanism. Prior to testing, the catalysts were reduced in H_2_ at 500 °C for 2 h. After reduction, the system was cooled to 220 °C under N_2_ flow to collect the background spectrum. Figure 5a–c show the DRIFTS spectra of the Ni/CeCrO_x_ catalyst at 220 °C under CO_2_, H_2_, and CO_2_/H_2_ atmospheres, respectively. Under a CO_2_ flow (Figure 5a), the spectra for 85 wt% Ni/CeCrO_x_ show characteristic bands for surface formate (1590 cm^−1^), methoxy (1082 and 2845 cm^−1^), linear CO (2035 cm^−1^), and bridged CO (1917 cm^−1^) [48,49,50]. The intensity of these bands gradually increases over time. Surface hydroxyl groups (OH) are observed in the 3600–3750 cm^−1^ region. A weak CH_4_ signal (3015 cm^−1^) is also detected under CO_2_ [51], suggesting that surface OH species spontaneously participate in the methanation reaction. When the gas flow is switched to H_2_ (Figure 5b), the surface hydroxyl groups (3600–3750 cm^−1^) are rapidly consumed, accompanied by a simultaneous increase in methoxy and CH_4_ signals [52]. Concurrently, the formate (HCOO) band intensity decreases. This indicates that CH_4_ is formed via the hydrogenation and dehydration of HCOO to CH_3_O, followed by further hydrogenation. The linear CO band disappears immediately after switching to H_2_, followed by the emergence of a CH_4_ signal, suggesting that CH_4_ can also be generated via the hydrogenation of linear CO. Under a CO_2_/H_2_ flow (Figure 5c), the bands corresponding to formate, methoxy, linear CO, and bridged CO increase in intensity over time. Notably, the CH_4_ signal intensity is significantly higher than that observed for 85 wt% Ni/Cr_2_O_3_.

For the Ni/Cr_2_O_3_ catalyst, the evolution of carbon species differs. Under CO_2_ (Figure 5d), characteristic bands for carboxylate (1620 and 1280 cm^−1^), methoxy (1082 and 2845 cm^−1^), linear CO (2035 cm^−1^), and bridged CO (1920 cm^−1^) are observed, with their intensities increasing over time [48]. Surface hydroxyl groups (3600–3750 cm^−1^) and a very weak CH_4_ signal (3015 cm^−1^) are also present. The intensities of the linear and bridged CO bands are notably higher on Ni/Cr_2_O_3_ than on Ni/CeCrO_x_. Upon switching to H_2_ flow, the bridged CO* (1920 cm^−1^) and linear CO (2035 cm^−1^) bands adsorbed at the Cr_2_O_3_-Ni interface disappear immediately, accompanied by CH_4_ (3015 cm^−1^) formation (Figure 5e,f). This clearly demonstrates that CH_4_ is primarily generated via the CO pathway on the 85 wt% Ni/Cr_2_O_3_ catalyst.

The intensity changes of CO species observed in both catalyst systems upon switching to H_2_ confirm that this intermediate forms from the further hydrogenation of HCOO/COOH species. Based on the spectroscopic evidence, it can be concluded that the formate pathway dominates on the Ni/CeCrO_x_ catalyst, whereas the reaction primarily follows the CO pathway on the Ni/Cr_2_O_3_ catalyst. For the Ni/CeCrO_x_ composite catalyst, the CO_2_ methanation proceeds via a dual pathway involving both formate and CO intermediates. This synergistic mechanism facilitates more efficient CO_2_ activation and hydrogenation, thereby explaining its superior catalytic performance in CO_2_ methanation.

## 3. Materials and Methods

### 3.1. Chemical Reagents

Nickel nitrate hexahydrate (Aladdin, shanghai, China, Ni(NO_3_)_2_·6H_2_O, AR 98%); cerium nitrate hexahydrate (Aladdin, shanghai, China, Ce(NO_3_)_3_·6H_2_O, AR 98%); chromium nitrate nonahydrate (Aladdin, shanghai, China, Cr(NO_3_)_3_·9H_2_O, AR 99%); magnesium oxide (Aladdin, shanghai, China, MgO, 99% metals basis); urea (Macklin, shanghai, China, CH_4_N_2_O, 99.9% metals basis); ethylene glycol (Aladdin, shanghai, China, C_2_H_6_O_2_, >99% (GC)); ethanol absolute (Aladdin, shanghai, China, CH_3_CH_2_OH, water ≤ 0.3%). All the above-mentioned reagents were used without further purification.

### 3.2. Preparation of Ni(OH)_2_ Nanosheets

Nickel nitrate hexahydrate (14.5 g) was dissolved in 100 mL of deionized water to form a homogeneous solution. Magnesium oxide powder (1.6 g) was subsequently introduced as an alkali source under continuous magnetic stirring, resulting in a uniform suspension. This mixture was maintained at 30 °C and stirred at 700 rpm for 48 h. During this period, a light-green precipitate gradually formed, indicating the successful generation of the nickel hydroxide product. The precipitate was collected via vacuum filtration and thoroughly rinsed with deionized water to remove residual ions. The obtained solid was then dried overnight in an oven at 100 °C. The final product was NiOH nanosheets.

### 3.3. Preparation of Catalysts

Nickel hydroxide nanosheets were ground into a fine powder to serve as the precursor. This powder was combined with cerium nitrate hexahydrate and chromium nitrate nonahydrate in 80 mL of a mixed solvent consisting of ethylene glycol and deionized water (9:1 *v*/*v*). Urea was introduced as a precipitating agent at a molar ratio of approximately 2:1 relative to the total metal ions. The resulting mixture was stirred magnetically at room temperature for 1 h, ensuring complete dissolution and the formation of a homogeneous solution. This solution was then transferred into a 100 mL Teflon-lined stainless-steel autoclave. The autoclave was sealed and maintained at 160 °C for 2 h to undergo hydrothermal treatment. After the reaction, the autoclave was allowed to cool naturally to room temperature. The solid precursor was collected and washed repeatedly with deionized water until the washings reached a neutral pH. It was subsequently washed with absolute ethanol via four cycles of centrifugation and redispersion to remove organic residues. The purified product was dried in an oven at 70 °C overnight. The dried material was ground into a fine powder and subjected to a two-step calcination process in air: first at 250 °C for 2 h, followed by 500 °C for another 4 h. This thermal treatment yielded the final catalyst. The actual loadings of nickel, cerium, chromium, and their corresponding oxides in the catalyst were quantitatively determined using inductively coupled plasma optical emission spectrometry (ICP-OES). Prior to evaluation in the CO_2_ methanation reaction, the catalyst powder was activated through in situ reduction in a pure hydrogen atmosphere at 500 °C for 2 h.

### 3.4. Catalytic Performance Evaluation

CO_2_ methanation activity was assessed in a fixed-bed quartz reactor operating under atmospheric pressure. In a typical experiment, 125 mg of catalyst was loaded into the reactor. Prior to reaction, the catalyst was pre-reduced under a pure H_2_ flow at 500 °C for 2 h. After reduction and cooling to reaction temperature, a reactant gas mixture (CO_2_:H_2_:N_2_ = 8:32:10) was introduced at a total flow rate of 50 mL min^−1^.

To assess temperature-dependent activity, the reaction temperature was gradually varied from 110 to 220 °C. The outlet gases were analyzed online using a gas chromatograph (Panna A91 Plus, Panna Beijing Panna Analytical Instruments Co., Ltd., Beijing, China) equipped with a thermal conductivity detector. CO_2_ conversion, CH_4_ selectivity, and CH_4_ space-time yield were calculated based on the chromatographic results. The calculation formulas are as follows:

$$Conv.\left(CO_{2}\right)\%=\frac{F(CO_{2})\times C_{in}(CO_{2})-FCO_{2}\times \frac{A_{N_{2}}-in}{A_{N_{2}}-out}\times C_{out}(CO_{2})}{F(CO_{2})\times C_{in}(CO_{2})}$$

$$Sel.\left({CH}_{4}\right)=\frac{n{CH}_{4}}{nCO+n{CH}_{4}}$$

$$STY({CH}_{4})(g_{{CH}_{4}}g_{cat}^{-1}h^{-1})=\frac{F\left({CO}_{2}\right)\times Conv.\left({CO}_{2}\right)\times Sel.({CH}_{4})\times 32\times 60}{22.4\times m_{cat}}$$

In these equations, *F* represents the gas flow rate, *C* the gas-phase concentration, *A* the corresponding chromatographic peak area, *m* the mass of catalyst, and *n* the molar quantity of the substance.

The carbon balance in the reaction process was calculated based on the CO_2_ methanation reaction. Firstly, the composition of the main product stream was analyzed using an online gas chromatograph (GC). Then, the measurement was repeated by recombining the separated secondary stream with the main product stream, and the composition of the mixed stream was fully analyzed using an online GC. The carbon balance data were calculated using the following equation:

$$C\;balance\left(\%\right)=\;\frac{C{O_{2}}_{out}+\;C{H_{4}}_{out}+\;CO_{out}\;+{\;2C}_{2}{H_{4}}_{out}\;+\;3C_{3}{H_{8}}_{out\;}}{{{CO}_{2}}_{in}}\times 100\%$$

Here, CO_2_ out, CH_4_ out, CO out, C_2_H_4_ out, C_3_H_8_ out, and CO_2_ in are the concentrations of the corresponding species in the reaction mixture determined by GC analysis. The subscripts ‘out’ and ‘in’ denote the final and initial concentrations, respectively. The numeric coefficient represents the number of carbon atoms in the given compound.

### 3.5. Structural Characterization

The elemental composition of the catalysts was determined using an Agilent 5110 inductively coupled plasma optical emission spectrometer (ICP-OES). Prior to analysis, catalyst powders were precisely weighed and transferred into polytetrafluoroethylene (PTFE) digestion vessels. A concentrated acid mixture was added, and the vessels were sealed and subjected to microwave-assisted digestion to ensure complete dissolution of the solid components. After cooling to room temperature, the digested solutions were diluted to a defined volume using deionized water in plastic volumetric flasks. The resulting solutions were analyzed sequentially. When necessary, further dilution was performed to ensure that analyte concentrations fell within the linear calibration range of the instrument.

XRD patterns were recorded using a Rigaku Ultima IV diffractometer (Rigaku Corporation, Tokyo, Japan) equipped with a Cu Kα radiation source (λ = 0.1541 nm), operated at 40 kV and 40 mA. Data were collected in continuous scan mode over a 2θ range of 10–80°, with a step size of 0.02° and a scan rate of 2° min^−1^. A 1° divergent slit and a 10 mm horizontal/longitudinal slit were employed. Both the scattering and receiving slits were fully open during measurements, and no Kβ filter was applied.

All samples were analyzed in powder form. The average crystallite size of metallic Ni was estimated using the Scherrer equation based on the full width at half maximum (FWHM) of the Ni (111) diffraction peak.

XPS measurements were performed on a Thermo Scientific K-Alpha spectrometer (Thermo Fisher Scientific, Waltham, MA, USA) equipped with a monochromatic Al Kα X-ray source (hν = 1486.6 eV). The chamber pressure during acquisition was maintained at ~5.0 × 10^−7^ mbar. The X-ray spot size was set to 400 μm, with an operating voltage of 12 kV and a filament current of 6 mA. Survey spectra were acquired at a pass energy of 150 eV with a step size of 1.0 eV, while high-resolution spectra were collected at 50 eV pass energy and 0.1 eV step size. Prior to analysis, a small amount of catalyst powder was pressed into a pellet and mounted onto the sample holder. Once the load-lock pressure dropped below 2.0 × 10^−7^ mbar, the sample was transferred into the analysis chamber. All binding energies were referenced to the C 1s peak at 284.6 eV to correct for charging effects.

In situ DRIFTS measurements were performed with a spectral resolution of 4 cm^−1^, with each scan acquired over ~1 min. Prior to data collection, background spectra were recorded under a N_2_ atmosphere at the designated test temperature. All spectra acquired during the reaction were normalized to the background and converted to Kubelka–Munk units for qualitative and semi-quantitative analysis. Once the target temperature was reached, a CO_2_/H_2_/N_2_ gas mixture (15.2:60.6:24.2, total flow rate = 50 mL min^−1^, atmospheric pressure) was introduced into the reaction cell. For separate adsorption experiments, the system was purged with N_2_ for 20 min after H_2_ exposure before CO_2_ was introduced. DRIFTS spectra were continuously collected over 60 min to enable time-resolved monitoring of surface-adsorbed intermediates. Characteristic vibrational bands corresponding to carbonate, formate, carboxylate, and hydroxyl species were analyzed to gain mechanistic insights into the CO_2_ hydrogenation process.

High-resolution transmission electron microscopy (HRTEM) was performed using a JEOL JEM-2100F microscope (JEOL Ltd., Akishima, Tokyo, Japan) equipped with a Schottky field emission gun and operated at an accelerating voltage of 200 kV. The instrument provides a point resolution of 0.23 nm and a lattice resolution of 0.14 nm, allowing for atomic-scale imaging. In scanning TEM (STEM) mode, high-angle annular dark-field (HAADF) imaging was carried out using the built-in HAADF detector, enabling Z-contrast imaging for distinguishing heavy-element distributions. Elemental mapping was conducted by energy-dispersive X-ray spectroscopy (EDS) using an Oxford Instruments X-Max silicon drift detector (Oxford Instruments, Abingdon, Oxfordshire, UK) (SDD) with an 80 mm^2^ active area. All samples were dispersed in ethanol, drop-cast onto standard copper TEM grids, and dried under ambient conditions prior to analysis. Multiple crystallographic orientations were observed to characterize nanostructure morphology and interface distribution.

CO_2_-TPD measurements were carried out on a BSD-Chem C200 automated chemisorption analyzer (Beishi Instruments, Beijing Beishi Technology Co., Ltd., Beijing, China). Approximately 100 mg of catalyst was first pretreated under a flow of pure He (50 sccm) at 200 °C for 60 min to remove surface impurities. After cooling to 50 °C, CO_2_ adsorption was conducted by exposing the sample to a CO_2_/He gas mixture (5 sccm CO_2_ and 45 sccm He; CO_2_:He = 1:9) at atmospheric pressure for 60 min. The system was then purged with He (50 sccm) for 40 min to eliminate weakly adsorbed (physisorbed) CO_2_. Desorption was performed by heating the sample from 50 °C to 600 °C at a ramp rate of 10 °C min^−1^ under continuous He flow. CO_2_ desorption was monitored in real time using a thermal conductivity detector (TCD, Agilent Technologies, Santa Clara, CA, USA). The resulting desorption profiles (TCD signal vs. temperature) were analyzed to assess the distribution and strength of basic sites on the catalyst surface.

H_2_-TPR experiments were performed using a BSD-Chem C200 automated chemisorption analyzer (Beishi Instruments). Approximately 30 mg of catalyst powder was loaded into a quartz reactor tube and pretreated under a helium flow (50 sccm) by heating to 200 °C at a rate of 10 °C min^−1^ and holding for 60 min to remove surface-adsorbed impurities and moisture. The sample was then cooled to 50 °C, and the gas feed was switched to 10% H_2_ in Ar (H_2_:Ar = 1:9, total flow rate = 50 sccm). To ensure uniform H_2_ distribution and surface interaction, the system was preheated from 50 °C at 2 °C min^−1^ for 30 min. The main TPR procedure was then conducted by ramping the temperature to 600 °C at 10 °C min^−1^ under the same H_2_/Ar atmosphere. Hydrogen consumption was monitored in real time using a thermal conductivity detector (TCD). The resulting TPR profiles were analyzed to assess the reducibility and interaction strength of various Ni species within the catalyst.

## 4. Conclusions

A highly active, selective, and thermally stable composite structured catalyst was successfully prepared via an ion-exchange method. Featuring inverse Ni/CeCrO_x_ composite active sites supported on nickel, the catalyst demonstrates excellent performance in the CO_2_ hydrogenation to methane reaction. The formation of CeCrO_x_ mixed oxides on the nickel surface significantly enhances the concentration of oxygen vacancies required for CO_2_ activation. In situ DRIFTS results reveal that both the formate pathway and the CO pathway coexist over the Ni/CeCrO_x_ catalyst. Compared to catalysts containing only a single type of interface, the composite oxide interface markedly promotes methane generation through synergistic effects. The inverse composite structure remains intact even after prolonged operation, thereby overcoming the inherent stability challenges of conventional supported catalysts. This work provides an innovative strategy for constructing highly stable, low-cost, and practical catalysts for CO_2_ methanation.

## Figures and Tables

**Table 1:** Physicochemical characteristics of the inverse catalysts.

Samples	Ni Loading (wt%)	Cr Loading (wt%)	Ce Loading (wt%)	S_BET_ (m^2^/g)	D_ads_ (nm)
85 wt% Ni/CeCrO_x_	21.2	2.1	2.0	149.9	21.4
85 wt% Ni/CeO_2_	19.9	0	4.0	107.6	14.1
85 wt% Ni/Cr_2_O_3_	19.6	3.9	0	101.9	19.3

## Data Availability

The data presented in this study are available on request from the corresponding author (please specify the reason for restriction, e.g., the data are not publicly available due to privacy or ethical restrictions).

## Supplementary Materials

The following supporting information can be downloaded at: https://www.mdpi.com/article/10.3390/ijms27073193/s1.
