# Supplementary material for: Inverse Ni/CeCrOx Catalysts for Enhanced Low-Temperature CO2 Methanation
Source: Int J Mol Sci. 2026 Mar 31;27(7):3193. doi: 10.3390/ijms27073193 (PMC13072776; doi:10.3390/ijms27073193)
Supplement: Supplementary file 1 [file ijms-27-03193-s001.zip › ijms-4225494-supplementary.pdf]

Supporting Information

Inverse Ni/$CeCrO_x$ Catalysts for Enhanced Low-Temperature CO_2 Methanation

Da Zhang, Haiyu Qi, Bowen Lei, Xuan Guo * and Feiyan Fu *

State Key Laboratory of Chemistry for NBC Hazards Protection, Beijing 102205, China; fireulc@163.com (D.Z.); haiyuqi2001@126.com (H.Q.); lbw3180@163.com (B.L.)

* Correspondence: guoxuan1010@126.com (X.G.); fufeiyan1@126.com (F.F.)

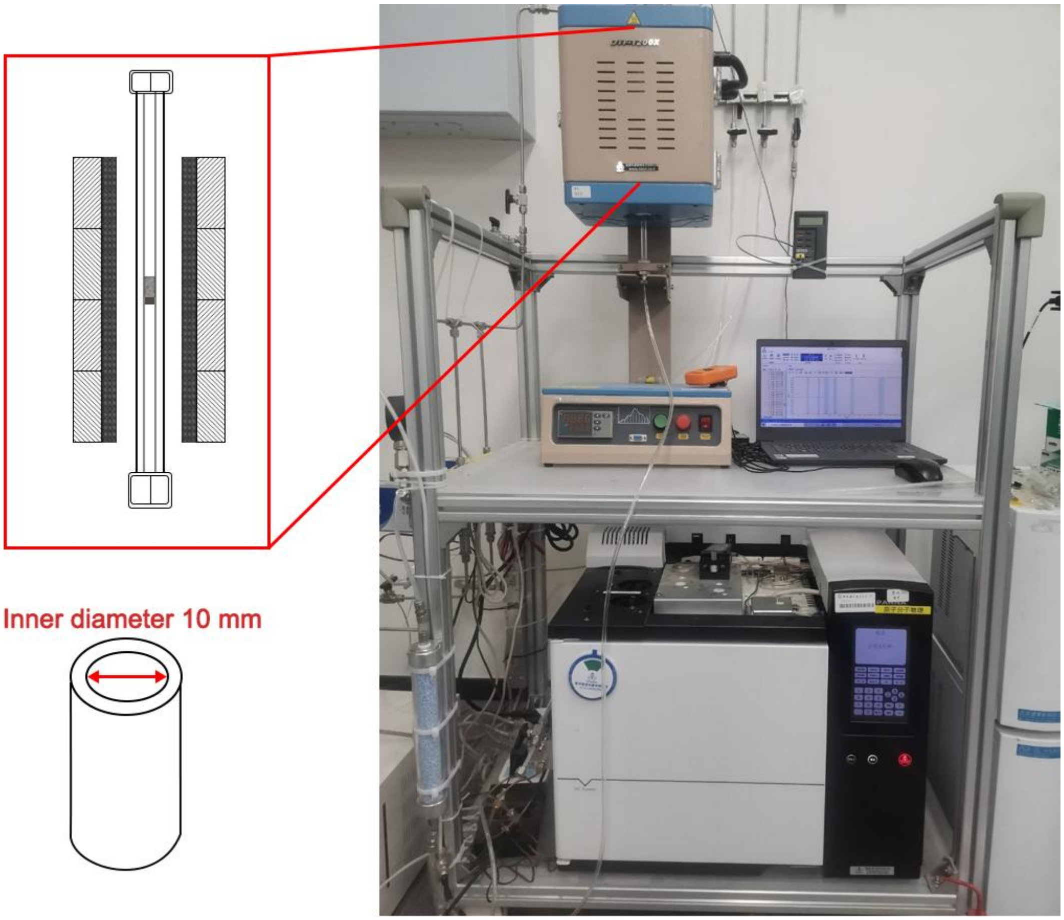

Figure S1. Schematic diagram of the activity evaluation reactor.

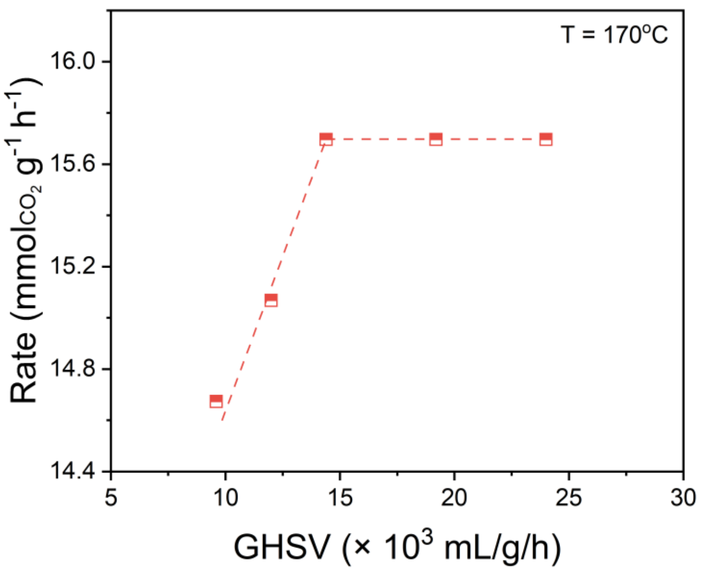

Figure S2. The results of the external diffusion experiment. Reaction condition: 16%CO_2/64%H_2/20%N_2, 0.1Mpa. Size = 0.425 mm and T = 165 °C.

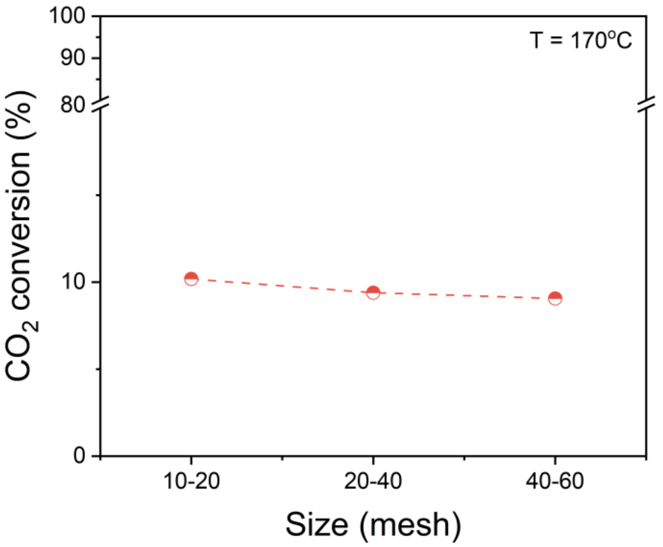

Figure S3. Influence of the mean catalyst size in the CO_2 conversion. Reaction condition: 16%CO_2/64%H_2/20%N_2, 0.1Mpa. 24,000ml/g/h and T = 165 °C.

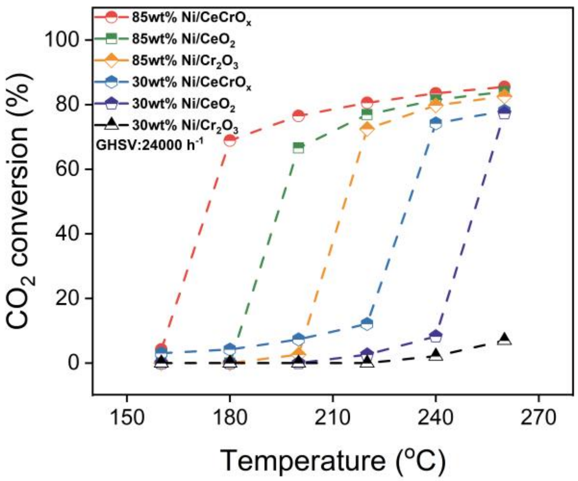

Figure S4. CO_2 conversion as a function of reaction temperature for catalysts.

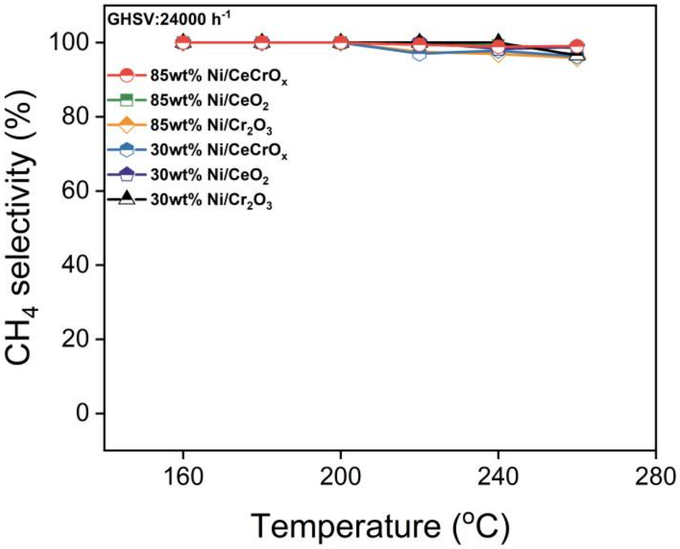

Figure S5. CH_4 selectivity as a function of reaction temperature.

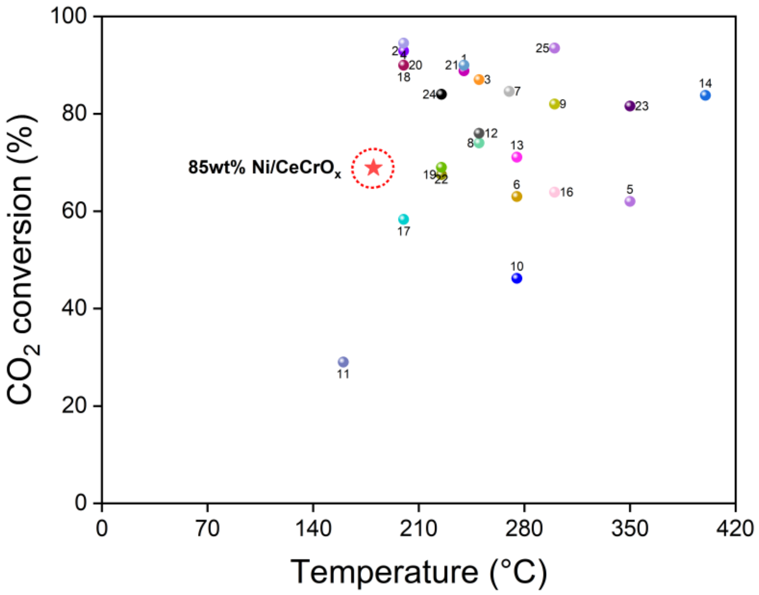

Figure S6. Comparison of CO_2 conversion performance with reported Ni-based catalysts for low-temperature CO_2 methanation.

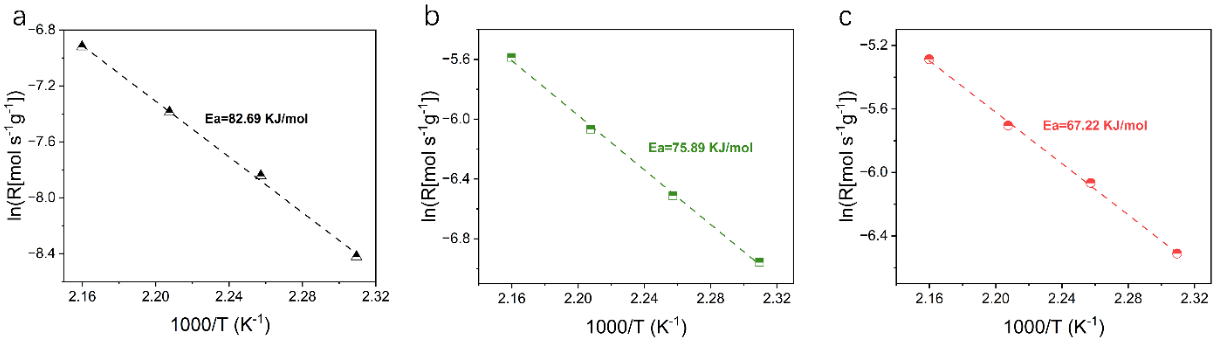

Figure S7. Ea calculations of different catalysts. (a) 85wt% Ni/$CeCrO_x$ catalyst. (b) 85wt% Ni/CeO_2 catalyst. (c) 85wt% Ni/Cr_2O_3 catalyst. (Reaction conditions for the catalytic test: WHSV=24000 mL/g_{cat}/h, CO_2: H_2: N_2=8:32:10, P=0.1 MPa)

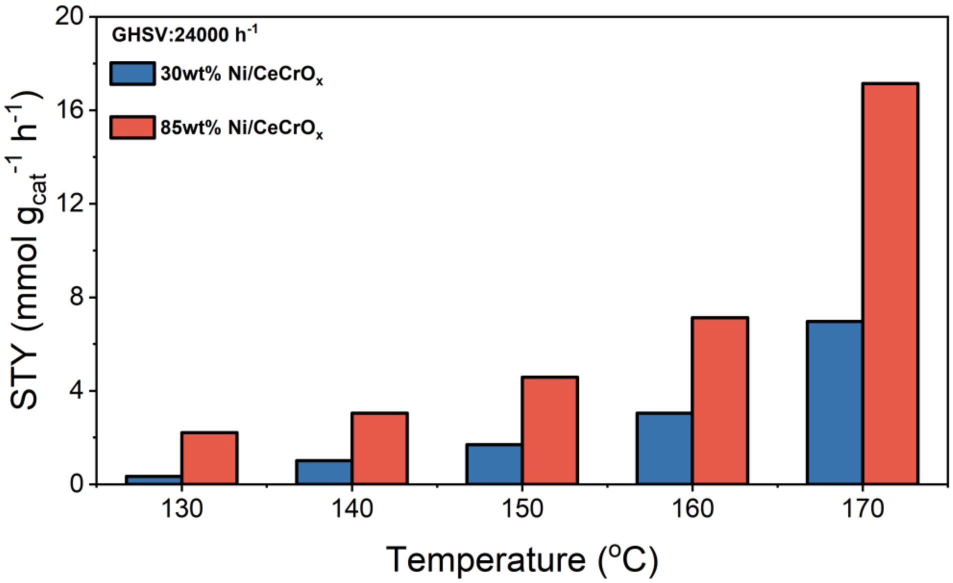

Figure S8. The STY of CH_4 as a function of 85wt% Ni/$CeCrO_x$ and 85wt% Ni/Cr_2O_3 catalysts, with CO_2 conversion <15%.

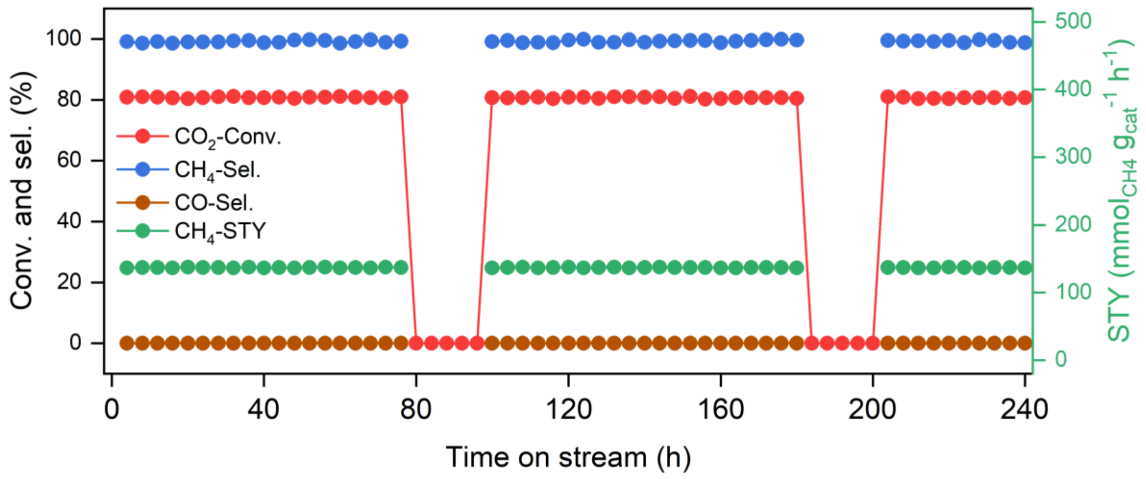

Figure S9. Stability test of 85wt% Ni/CeCrO$_x$ catalyst. (Reaction conditions for the catalytic test: catalyst 125mg, GHSV=24000mL/g_{cat}/h, 220°C, CO_2: H_2: N_2=8:32:10, total flow rate=50mL/min, P=0.1 MPa)

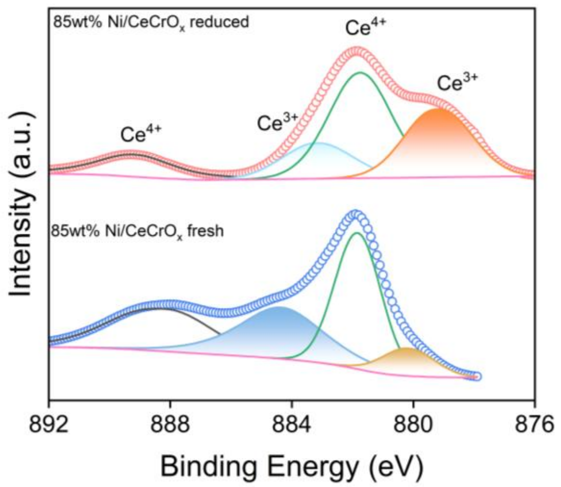

Figure S10. Ce3d XPS spectra of the fresh 85wt% Ni/$CeCrO_x$ catalyst and the reduced 85wt% Ni/$CeCrO_x$ catalyst.

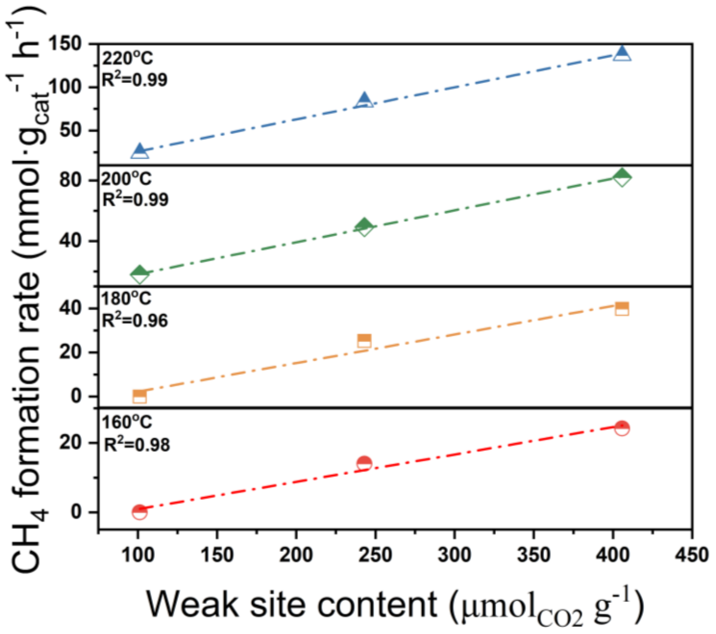

Figure S11. Relationship between the reaction rate and weak site content.

Table S1. A comprehensive comparison of this work with similar work published in the literature.

Catalyst	GHSV (mLg^{-1} h^{-1})	T (^oC)	P (MPa)	XCO_2 (%)	SCH_4 (%)	Ref
Ni/Ce-Al-0.1	48000	240	0.1	88.83	99.96	[1]
IZ2	15000	200	0.1	92.9	100	[2]
$Ni_{70}Al_{30}$	60000	250	0.1	87	100	[3]
NiMnN-LDO	30000	200	0.1	94.51	98.12	[4]
NT-5	30000	350	0.1	62	98	[5]
$Ni/Ce_{0.5}La_{0.5}O_2$	60000	275	0.1	63	48	[6]
$Ni/Ce_{0.9}Y_{0.1}O_\delta$	10000	270	0.1	84.6	99.8	[7]
Ni_7Co_3Al-R	30000	250	0.1	74	100	[8]
Ru/CeO_2-U	18000	300	0.1	82	100	[9]
1% Ru/DBD-ZrO_2	60000	275	0.1	46.2	100	[10]
Co-Al-O-550	1000	160	2	29	89.9	[11]
Ni/CeO_2-O_v-R	20000	250	0.1	76	100	[12]
8Ni-La/SiO_2	36000	275	0.1	71.1	99.6	[13]
$LaNiO_3/CeO_2$	30000	400	0.1	83.8	99	[14]
Ni-N	6000	300	0.1	92	100	[15]
CoZn-Z	37500	300	1atm	63.9	99.2	[16]
20Ce80Co	--	200	0.1MPa	58.3	90	[17]
15 mol% ZrO_2/Ni	60000	200	0.1MPa	~90	99.4	[18]
CeO_2/Ni-4	13500	225	0.1MPa	67.5	100	[19]
$CeZrO_x$/Ni	15000	200	1atm	90	99	[20]
$CeAlO_x$/Ni/Ni-foam	10000	240	0.1MPa	90	100	[21]
Ni/La0.2-$MgAlO_x$	48000	225	0.1	69.0	~98	[22]
Ni-CeO_2/hBFS	12000	350	0.1	81.6	99.8	[23]
Ni/CeO_2-EDA	20000	225	0.1	84	~100	[24]
Ni/MoCe	24000	300	0.1	93.5	100	[25]
85wt% Ni/$CeCrO_x$	24000	220	0.1MPa	80.6	99	This work
85wt% Ni/CeO_2	24000	220	0.1MPa	76.9	99	This work

85wt% Ni/Cr_2O_3	24000	200	0.1MPa	72.5	99	This work

Table S2. XPS analysis of Ni species present on the surface of fresh 85wt% Ni/$CeCrO_x$ and reduced 85wt% Ni/$CeCrO_x$.

Catalysts		Ni *2p*	
		**Ni^0**	**Ni^{2+}**
85wt% Ni/$CeCrO_x$ fresh	BE (eV)	852.2	854.8
	Area (eV)	13379.6	6714.0
	R_a	0.67	0.33
85wt% Ni/$CeCrO_x$ reduced	BE (eV)	853.5	855.6
	Area (eV)	52018.8	45889.3
	R_a	0.53	0.47

R_a= Relative area ratio

R_a = {(Individual area in Ni 2*p*)/(Sum of areas in Ni 2*p*)}

Table S3. XPS analysis of Ce species present on the surface of fresh 85wt% Ni/$CeCrO_x$ and reduced 85wt% Ni/$CeCrO_x$.

**Catalysts**		**Ce *3d***			
		**Ce^{4+}**	**Ce^{3+}**	**Ce^{4+}**	**Ce^{3+}**
85wt%	BE (eV)	888.2	884.3	881.8	880.2
Ni/$CeCrO_x$	Area (eV)	13687.6	14591.2	19272.9	4372.1
fresh	R_a	0.26	0.28	0.37	0.08
85wt%	BE (eV)	889.1	883.2	881.7	879.1
Ni/$CeCrO_x$	Area (eV)	6294.4	7881.3	21321.5	14503.6
reduced	R_a	0.13	0.16	0.43	0.29

R_a= Relative area ratio

R_a = {(Individual area in Ce 3*d*)/(Sum of areas in Ce 3*d*)}

Table S4. XPS analysis of Cr species present on the surface of fresh 85wt% Ni/$CeCrO_x$ and reduced 85wt% Ni/$CeCrO_x$.

Catalysts		Cr *2p*		
		**Cr^{3+}**	**Cr^{2+}**	**Cr^{3+}**
85wt% Ni/$CeCrO_x$ fresh	BE (eV)	585.79	576.98	575.50
	Area (eV)	15908.2	9110.7	10320.6
	R_a	0.45	0.26	0.29
85wt% Ni/$CeCrO_x$ reduced	BE (eV)	587.8	579.7	576.8
	Area (eV)	8715.4	16501.8	5865.4
	R_a	0.28	0.53	0.19

R_a= Relative area ratio

R_a = {(Individual area in Cr 2*p*)/(Sum of areas in Cr 2*p*)}

Table S5. XPS analysis of O species present on the surface of fresh 85wt% Ni/$CeCrO_x$ and reduced 85wt% Ni/$CeCrO_x$.

**Catalysts**		**O *1s***		
		**O_{OH}**	**O_{vac}**	**O_L**
85wt% Ni/$CeCrO_x$ fresh	BE (eV)	532.2	530.9	529.1
	Area (eV)	6088.9	25114.8	38763.1
	R_a	0.09	0.36	0.55
85wt% Ni/$CeCrO_x$ reduced	BE (eV)	532.6	531.1	530.1
	Area (eV)	5143.6	48947.0	26203.4
	R_a	0.06	0.61	0.33

R_a= Relative area ratio

R_a = {(Individual area in O 1*s*)/(Sum of areas in O 1*s*)}

Table S6. CO_2-TPD results of the catalysts after reduction.

Catalysts	CO_2 desorption ($\mu mol_{CO2}\ g_{cat}^{-1}$)			
	Weak (<200 °C)	Medium (200-400 °C)	Strong (>400 °C)	Total
85wt% Ni/CeCrOx	405.7	27.2	93.4	526.3
85 wt% Ni/CeO_2	243.1	46.3	319.5	608.9
85 wt% Ni/Cr_2O_3	101.2	88.9	37.2	227.3

References

[1] H. Fu, H. Lian, Optimizing low-temperature CO_2 methanation with aluminum-doped Ni/CeO_2 catalysts: insights into reaction pathway adjustments and strong metal-support interactions, Chemical Engineering Journal 489 (2024) 151021.
[2] H. Zhou, J. Yang, Y. Song, T. Geng, N. Zhao, Q. Wang, F. Xiao, J. Luo, The inverse configuration confers high activity and stability to ZrO_2/Ni catalysts for CO_2 methanation, Advanced Functional Materials 35(51) (2025) e25888.
[3] N.J. Martins, O.W. Perez-Lopez, Tuning the composition of Ni-Al-LDH catalysts for low-temperature CO_2 methanation, Fuel 381 (2025) 133594.
[4] D. Qiang, T. Mei, Y. Liu, H. Jin, Z. Ye, Z. Li, S. Zhao, Enhanced low-temperature activity for CO_2 methanation over N-doped NiMn-LDO, Chemical Engineering Journal 507 (2025) 160839.
[5] Y. Park, Y. Ju, N. Pal, D.Y. Kim, E.-B. Cho, S.B. Kang, Corrigendum to "Facile one-pot solvothermal synthesis of enlarged mesoporous nickel phyllosilicate spherical catalyst for CO_2 methanation"[J. Alloy. Compd. 1029 (2025) 180743](Journal of Alloys and Compounds (2025) 1029,(S0925838825023047),(10.1016/j. jallcom. 2025.180743)), (2025).
[6] P. Kaisook, P. Athikaphan, S. Nijpanich, T. Minato, S. Neramittagapong, A. Neramittagapong, Ni/CeO_2 catalyst with La and Zr additives for improved low-temperature CO_2 methanation efficiency, Results in Engineering 25 (2025) 103795.
[7] J. Zhang, L. Yuan, Y. Li, Y. Liang, L. Zhou, Y. Chen, Boosting the CO_2 methanation over Ni/Ce0. 90RE0. 10Oδ by regulating of oxygen vacancy density, Molecular Catalysis 579 (2025) 115040.
[8] F. Zhang, B. Lu, L. Xu, Outstanding low-temperature activity and stability of NiCo alloy catalysts derived from NiCoAl-LDHs for CO_2 methanation, Journal of the Energy Institute 120 (2025) 102070.
[9] Y. He, X. Zheng, D. Mao, T. Meng, H. Mao, J. Yu, Promoting catalytic CO_2 methanation using Ru catalyst supported on Ce-MOF-derived CeO_2, Renewable Energy 245 (2025) 122834.
[10] M. Liu, R. Zou, C.-j. Liu, Improvement in the activity of Ru/ZrO_2 for CO_2 methanation by the enhanced hydrophilicity of zirconia, Applied Catalysis B: Environment and Energy 360 (2025) 124549.

[11] W. Song, K. Wang, X. Wang, Q. Ma, T.-S. Zhao, J.W. Bae, X. Gao, J. Zhang, Boosting low temperature CO_2 methanation by tailoring Co species of CoAlO catalysts, Chemical Engineering Science 298 (2024) 120405.
[12] W. Yang, K. Chang, M. Yang, X. Yan, S. Yang, Y. Liu, G. Wang, F. Xia, H. Wang, Q. Zhang, Facilitating CO_2 methanation over oxygen vacancy-rich Ni/CeO_2: Insights into the synergistic effect between oxygen vacancy and metal-support interaction, Chemical Engineering Journal 499 (2024) 156493.
[13] X. Chen, Y. Zhang, C. Sun, Y. Wang, G. Song, C. Li, K.H. Lim, R. Ye, Y. Peng, H. Arandiyan, Lanthanum-mediated enhancement of nickel nanoparticles for efficient CO_2 methanation, Fuel 371 (2024) 131998.
[14] T. Zhang, J. Tian, Y. Zhou, J. Zeng, X. Sun, Z. Gong, Enhanced CO_2 methanation over $LaNiO_3$/CeO_2 derivative catalyst with high activity and stability, International Journal of Hydrogen Energy 71 (2024) 1081-1089.
[15] Y. Hu, Y. Men, S. Xu, Y. Feng, J. Wang, K. Liu, Y. Zhang, Modulating nickel precursors to construct highly active Ni/Y_2O_3 catalysts for CO_2 methanation, International Journal of Hydrogen Energy 81 (2024) 1311-1321.
[16] Y.-T. Li, L. Zhou, G.-N. Han, W.-G. Cui, W. Li, T.-L. Hu, ZIF-derived catalyst with inverse ZnO/Co structure for efficient CO_2 methanation, International Journal of Hydrogen Energy 51 (2024) 452-461.
[17] Y. Gao, V. Muravev, Y. Fan, H. Zhang, J. Wagemakers, A. Parastaev, N. Kosinov, E.J. Hensen, Strong Stabilization of Co Nanoparticles by CeO_2-x Clusters in Inverse CeO_x/Co Catalysts for Enhanced CO_2 Methanation, Advanced Materials (2025) e10593.
[18] X. Tang, Y. Wang, J. Zhang, C. Yu, M. Cheng, S. Yang, X. Yang, L. Liu, L. Han, Y. Xu, Spontaneous Nano-ZrO_2 Exsolution From Ni-Zr-O Mixed Oxides Enables Facile Fabrication of ZrO_2/Ni Inverse Catalysts for Efficient CO_x Methanation, Angewandte Chemie International Edition 64(36) (2025) e202511453.
[19] Y. Zang, T. Wei, J. Qu, F. Gao, J. Gu, X. Lin, S. Zheng, Boosting low-temperature CO_2 methanation activity through Tailored electronic structures and step-edge defects in Niε−-Ov-Ceδ+ inverse interfaces, Applied Surface Science 685 (2025) 161945.

[20] C. Song, J. Liu, R. Wang, X. Tang, K. Wang, Z. Gao, M. Peng, H. Li, S. Yao, F. Yang, Engineering MO_x/Ni inverse catalysts for low-temperature CO_2 activation with high methane yields, Nature Chemical Engineering 1(10) (2024) 638-649.
[21] X. Tang, C. Song, H. Li, W. Liu, X. Hu, Q. Chen, H. Lu, S. Yao, X.-n. Li, L. Lin, Thermally stable Ni foam-supported inverse $CeAlO_x$/Ni ensemble as an active structured catalyst for CO_2 hydrogenation to methane, Nature Communications 15(1) (2024) 3115.
[22] Z. Wang, T. Zhang, T.R. Reina, L. Huang, W. Xie, N.M. Musyoka, B. Oboirien, Q. Wang, Enhanced low-temperature CO_2 methanation over La-promoted NiMgAl LDH derived catalyst: Fine-tuning La loading for an optimal performance, Fuel 366 (2024) 131383.
[23] X. Chen, Y. He, X. Cui, L. Liu, High value utilization of waste blast furnace slag: New Ni-CeO_2/hBFS catalyst for low temperature CO_2 methanation, Fuel 338 (2023) 127309.
[24] R. Zhao, Y. Xie, Z. Li, H. Weng, D. Zhu, Y. Mao, H. Wang, Q. Zhang, Unveiling the promotion effect of ethylenediamine on preparation of Ni/CeO_2 catalyst for low-temperature CO_2 methanation, International Journal of Hydrogen Energy 51 (2024) 451-463.
[25] X. Zou, J. Liu, Y. Li, Z. Shen, X. Zhu, Q. Xia, Y. Cao, S. Zhang, Z. Ge, L. Cui, Molybdenum-doping promoted surface oxygen vacancy of CeO_2 for enhanced low-temperature CO_2 methanation over Ni-CeO_2 catalysts, Applied Surface Science 661 (2024) 160087.
